# Adulteration of the Herbal Weight Loss Products by the Illegal Addition of Synthetic Antiobesity Medications: A Pilot Study

**DOI:** 10.1155/2021/9968730

**Published:** 2021-07-14

**Authors:** Farzin Firozian, Amir Nili-Ahmadabadi, Shirin Moradkhani, Miad Moulaei, Zohreh Fasihi, Davoud Ahmadimoghaddam

**Affiliations:** ^1^Medicinal Plants and Natural Products Research Center, Hamadan University of Medical Sciences, Hamadan, Iran; ^2^Department of Pharmaceutics, School of Pharmacy, Hamadan University of Medical Sciences, Hamadan, Iran; ^3^Department of Pharmacology and Toxicology, School of Pharmacy, Hamadan University of Medical Sciences, Hamadan, Iran; ^4^Department of Pharmacognosy, School of Pharmacy, Hamadan University of Medical Sciences, Hamadan, Iran; ^5^Food and Drug Control Laboratory, Hamadan University of Medical Sciences, Hamadan, Iran

## Abstract

**Background:**

Some anorexic agents are used to fraudulent augmentation herbal weight loss formulations. This study was designed to evaluate the potential existence of illicit substances in 63 herbal weight loss formulations collected from local apothecaries in Hamadan, Iran.

**Methods:**

The thin-layer chromatography method was applied for the primary screening of potential illicit substances in the samples. The positive samples were analyzed using an isocratic high-performance liquid chromatography method.

**Results:**

The results showed that 26.98% of the samples contained 17.76 ± 6.02 mg/cap of sibutramine. Daily therapeutic dose intake of sibutramine is in the range of 5 to 15 mg daily.

**Conclusion:**

Since apothecaries have advised consumers to take at least two capsules a day, it seems that the blood concentration of sibutramine will likely rise beyond the therapeutic concentration and become toxic. Therefore, the usage of such products could pose serious risks to consumers' health.

## 1. Introduction

Nowadays, obesity has become a challenge in public health due to the increasing trend in its prevalence. Based on the World Health Organization's (WHO) definition of overweight and obesity, obesity is described as “abnormal or excessive fat accumulation that may impair a person's health” [1]. A meta-analysis and systematic review reported that the total prevalence rate of obesity in adults in Iran is 21.4% [2]. Obesity would increase the risk of developing various chronic diseases such as cancer, diabetes, psychosocial dysfunction, gout, arthritis, and cardiovascular disease. Overall, obesity increases the rate of morbidity and mortality. Considering these health risks as a consequence of obesity, weight loss and treatment of obesity are of high importance. Hence, various methods such as diet, physical activity, pharmacologic strategies, and surgical intervention have been used to lose weight.

Since the public believes that natural herbal compounds are safe and have no side effects, medicinal herbs are extensively used to maintain or improve health [3, 4]. Herbal weight loss supplements are a very popular and commonly used approach in order to lose weight in Iran and other countries [5]. All weight loss products must be approved by the Food and Drug Organization and manufactured based on Good Manufacturing Practice (GMP) methods. There is growing concern over the presence of illicit substances, such as CNS stimulants and psychotropic agents in these products that could bring serious adverse effects. Sibutramine, methamphetamine, metformin, and bupropion are the most illicit substances found in herbal weight loss products [6]. The addition of these medicines to herbal weight loss may result in many side effects such as dry mouth, hand tremors, hypertension, sleep disturbances, loss of appetite, palpitations, asthma, renal and liver damage, and cardiovascular diseases [7].

Therefore, the present study aimed to determine the potential illegal adulterant in herbal weight loss products supplied for weight loss in Hamadan, Iran.

## 2. Material and Methods

### 2.1. Sampling

This experimental study was conducted between December 2019 and July 2020 in Hamadan University of Medical Sciences, Hamadan, Iran. A total of 63 samples of available herbal weight loss products were randomly collected from local apothecaries in Hamadan. All samples were kept under suitable conditions and analyzed as soon as possible.

### 2.2. Apparatus and Reagents

Standard sibutramine hydrochloride (SH) and methamphetamine were obtained from Sigma-Aldrich Chemical Company (St. Louis, MO, USA). Metformin and bupropion were obtained as a gift from Raha Pharmaceutical Company, Tehran, Iran. All other solvents and chemicals with a purity of 99% (HPLC grade) were obtained from Merck, Darmstadt, Germany.

The high-performance liquid chromatography (HPLC) method was utilized to quantitatively analyze sibutramine in herbal samples. For this purpose, an HPLC system (LC20AD XR, Shimadzu Company) equipped with a photodiode array detector (PDA) was used.

### 2.3. Sample Preparation

The samples (50 mg) were weighed and soaked in a solvent consisted of methanol: water in an 80 : 20 proportion. Then, samples were filtered through Whatman filter paper (No. 1) and placed in a rotary evaporator under vacuum at 60°C and 90 RPM. The whole process was repeated three times. The samples were kept in the refrigerator until the time of analysis.

### 2.4. Qualitative Detection of Illicit Substances in Herbal Formulations

The thin-layer chromatography (TLC) method was applied to the qualitative detection of illicit substances, such as sibutramine, methamphetamine, metformin, and bupropion, in the samples. A TLC-sheet coated with silica gel was used as the stationary phase. To detection of sibutramine and methamphetamine, the mobile phase was prepared from methanol: ammonia (100 : 1.5). In addition, the solvent system for detection of metformin and bupropion was glacial acetic acid, butanol, and water (10 : 40 : 50), and methanol and ammonium (90 : 10), respectively. A spot of standard and sample was applied on the bottom edge of the TLC plate (at about 1.5 centimeters from the bottom edge) using a capillary tube. Finally, the TLC plate was placed in the chamber containing mobile phase to separate the spots and visualized using ultraviolet light (wavelength: 245 nm) and Dragendorff's reagent.

### 2.5. HPLC Analysis Method

An isocratic HPLC method was developed for the analysis of sibutramine in herbal formulations. Reversed-phase C18 (PerfectSil Target ODS-3, MZ-Analysentechnik GmbH, Germany) HPLC column was used for this purpose. The wavelength of the detector was set at 225 nm. The mobile phase consisted of analytical grade acetonitrile (Merck, Germany) and phosphate buffer (40 : 60; pH = 6.2). The flow rate was 1 mL/min. About 20 *μ*L of each sample was injected into the HPLC system, and the runtime for each injection was considered 10 minutes.

The limit of detection (LOD) and the limit of quantification (LOQ) were calculated using the ratio of signal/noise = 3 and signal/noise = 10, respectively [8]. The recovery experiments were performed as well.

## 3. Result and Discussion

The present study was carried out aiming to detect and quantify the adulteration of herbal weight loss products. Based on TLC analysis, none of the samples contained methamphetamine, bupropion, and metformin (Table 1). The TLC analysis demonstrated that 17 of the samples (26.98%) were contained sibutramine.

To the quantitative analysis of sibutramine using the HPLC method, a calibration curve was drawn and shown that the calibration curve in the *r*^*2*^ index was within the acceptable linear range. The LOD and LOQ were determined to be 1.17 and 3.56 ng/mL, respectively (Table 2). Furthermore, the recovery tests were shown that the results are in an acceptable range (92.5–106.8%). Consequently, the recovery tests are indicating the accuracy and precision of the analytical methods used in this study.

Previously, Ariburnu et al. compared HPLC and high-performance thin-layer chromatography (HPTLC) methods for sibutramine analysis. They revealed that both methods were useful for the routine analysis of illegally added sibutramine in the marketed products. However, the HPLC method is related to a better recovery rate and lower probability of tailing [9].

In the present study, the positive samples from TLC screening were subjected to the HPLC analysis. As shown in Table 3, the minimum and maximum of sibutramine content in positive samples were 4.38 and 26.37 mg/capsule (cap). In addition, the mean value of sibutramine in the positive samples was detected as 17.76 ± 6.02 mg/cap. In a previous study by Mathon et al., a total of 39 weight loss supplements were screened. They reported that 17 out of 39 weight loss supplements contained sibutramine ranging from 3 to 35 mg/cap as an adulterant [10]. In addition, other studies showed that the sibutramine range in some of the adulterated herbal products was about 2 to 26 mg per single dosage [11, 12].

Sibutramine, a serotonin-noradrenaline reuptake inhibitor, is known as a central anorectic that promotes and maintains weight loss in obese people. It has several side effects such as nervousness, xerostomia, headache, numbness, paresthesia, and cardiovascular diseases. Hence, it has been withdrawn from the market. Daily therapeutic dose intake of sibutramine is in the range of 5 to 15 mg daily [13]. Since apothecaries have advised consumers to take at least two capsules a day, it seems that the blood concentration of sibutramine will likely rise beyond the therapeutic concentration and become toxic. Therefore, the usage of such products could pose serious risks to consumers' health.

## 4. Conclusion

Taken together, the presence of synthetic drugs, such as sibutramine in herbal weight loss products is illegal; however, this compound might be added to these products in order to reach higher efficacy and lower cost. The present study confirmed the existence of sibutramine as an adulterant in some herbal weight loss products which might cause serious and life-threatening adverse effects.

## Figures and Tables

**Table 1:** Preliminary examination on samples using the TLC method.

Drugs	Total samples	Positive samples	%
Methamphetamine	63	ND	ND
Sibutramine	63	17	26.98
Metformin	63	ND	ND
Bupropion	63	ND	ND

ND: none detected.

**Table 2:** Linearity range, detection limits, and summary of method validation data for HPLC analyses.

Drug	Range (ng/mL)	Linear equation	*r* ^2^	LOD (ng/mL)	LOQ (ng/mL)	Recovery (%)	RSD (%)
Sibutramine	5–200	*Y* = 36358*x* + 123.12	0.999	1.17	3.56	101.36	2.67

**Table 3:** The sibutramine level and its daily intake through consumption of herbal medicine.

Drug	Drug concentration (mg/cap)			Daily intake (mg/kg body weight)		
	Mean (mg/cap)	Minimum (mg/cap)	Maximum (mg/cap)	Mean ± SD	Minimum	Maximum
Sibutramine	17.76 ± 6.02	4.38	26.37	35.53 ± 12.23	8.76	52.74

## Data Availability

The data used to support the findings of this study are included within the article.
